# Non-Communicable Diseases Impact Low-Income Households in Malaysia

**DOI:** 10.21315/mjms2024.31.1.11

**Published:** 2024-02-28

**Authors:** Norfatihah Isamail, Rusmawati Said, Normaz Wana Ismail, Sharifah Azizah Haron

**Affiliations:** 1 School of Business and Economics, Universiti Putra Malaysia, Selangor, Malaysia; 2 Office of Deputy Dean (Thesis, Student Affairs and Media), School of Gradute Studies, Universiti Putra Malaysia, Selangor, Malaysia; 3 Office of Deputy Dean (Research, Funding, Corporate and Community Linkages), UPM School of Business and Economics, Selangor, Malaysia; 4 Department of Resource Management and Consumer Studies, Faculty of Human Ecology, Universiti Putra Malaysia, Selangor, Malaysia

**Keywords:** non-communicable disease, catastrophic health expenditure, poverty, Malaysia

## Abstract

**Background:**

Non-communicable diseases (NCDs) have a vast and rising impact on households at all income levels across the globe, particularly with poorer people bearing the burden. Hence, this study examines NCDs’ effects on Malaysia’s B40 group (low-income earners).

**Methods:**

This study used the 2015 National Health and Morbidity Survey, a population-based cross-sectional survey with 18,616 respondents from B40 households in Malaysia. Logistic regression analysis is used to assess NCDs’ influence on poverty.

**Results:**

In 2015, more than 20% of the B40 households lived below the poverty level. In addition, the B40 households had a greater prevalence of NCDs, with almost half of them diagnosed with at least one NCD (47.32%); hypertension (9.90%), diabetes mellitus (17.12%) and hypercholesterolemia (22.89%). Households with a member having an NCD are more likely to experience poverty than those without NCDs. The results also suggested that B40 households with catastrophic payments were at a 25% threshold; the elderly, individuals without formal education and unpaid workers were more likely to experience poverty.

**Conclusion:**

The findings suggest that NCDs increase the likelihood of B40 households falling into poverty. These facts highlight the necessity of safeguarding B40 households from the financial burden of NCDs by creating more effective financial protection plans for Malaysia’s low-income earners.

## Introduction

Non-communicable diseases (NCDs) can have tremendous economic repercussions on low-income households. An NCD is a chronic illness that cannot be transmitted to others and has a lasting impact on a person’s life (1). It is brought on by hereditary, physiological, environmental and behavioural variables (1). The four most common NCDs are cardiovascular diseases, heart disease and stroke, cancer, diabetes and chronic respiratory (2). According to the 2015 National Health Malaysian Survey (NHMS) (3), NCDs are becoming widespread and likely responsible for 73% of deaths in Malaysia. The prevalence of NCDs in Malaysia keeps rising, with 11.6% and 20.7% in 2006, rising to 18.3% and 38.1% in 2019, attributable to diabetes and hypercholesterolemia, respectively (4–6). Furthermore, almost half of the B40 population (47.6%) have at least one undiagnosed NCD (3).

The epidemic of NCDs is rapidly spreading and is responsible for more than 70% of global deaths. They are also accountable for more than 50% of the worldwide disease burden (7). Non-communicable diseases can potentially impose enormous and unwarranted personal, societal and economic costs, leading to the impoverishment of families, the strain on healthcare systems and economic damage to nations (8, 9). The increasing demand for medical needs in Malaysia is straining the country. Malaysia’s high prevalence of NCDs burdens the healthcare system. In addition, NCDs can also have tremendous economic repercussions on the household, particularly among low-income earners. The World Health Organization (WHO) (2) 2022 report is a stark reminder of how big a threat NCDs are. Based on this report, the Malaysian government must have the right policies to prevent and treat NCDs.

The Ministry of Health (MOH) Malaysia (10) reports on selected NCDs for estimated direct healthcare costs, which include cardiovascular disease, diabetes and cancer. The higher costs of NCDs are primarily accounted for by primary care, outpatient attendance, and routine diagnostic and monitoring tests. The more complex tests, such as an echocardiogram and coronary angiogram, with larger amounts captured in hospitalisation costs, were primarily performed in the inpatient settings (10). The MOH Malaysia reported that the prevalence of hypertension and diabetes is the highest and fastest rising among B40 households (5, 11). Low-income earners are disproportionately affected by NCDs, which suggests a correlation between poverty and NCDs. Psychological stress, the high rate of environmentally induced risky behaviours, and restricted access to high-quality and affordable healthcare increase the susceptibility of less affluent people.

The relationship between poverty and NCDs extends far beyond healthcare provisions. In most high-income countries, people with NCDs are covered by social security and discretionary financial ability, allowing them to afford the necessary healthcare. In low and middle-income countries, existing health insurance systems are inadequate or non-existent. As a result, NCD care expenses are handled personally, which usually has catastrophic effects or treatment is foregone. The current study will provide evidence that can contribute to better policy implementations to assist authorities in providing an insurance package with comprehensive benefits while introducing a more sustainable way to supplement existing health financing. A National Health Insurance (NHI) programme that the government manages is one of the potential financing mechanisms for the B40s. It can also provide social protection for these households.

Most countries have implemented the NHI, which enhances access to care and health systems. Taiwan and Thailand have successfully managed health expenditure expansions using NHI systems. This approach ensures that the same extensive benefits package covers everyone, that all healthcare providers are paid at the same rates, and they provide access to private and public healthcare. The NHI protects households from NCDs’ potentially catastrophic effects and promotes the general population’s health, especially impoverished persons. Therefore, it is essential to comprehend and provide information in this area; the relationship between NCDs and low-income households. The information will enable the government and policymakers to act and emphasise the need to adopt policies and actions to safeguard society from NCD threats.

## Methods

### Study Design and Sampling

This study used secondary data from the NHMS 2015. Volume III of the 2015 NHMS was carried out by the Institute of Public Health (IPH) within MOH Malaysia. The NHMS volume III 2015 survey data are nationally representative and the first on new cycles. NHMS 2015 employed a two-stage stratified random sampling method to ensure national representativeness (12). Malaysia’s states and federal territories are considered part of the primary stratum, while the urban and rural areas are considered part of the secondary stratum (12). Primary sampling units (PSUs) were enumeration blocks (EBs) and secondary sampling units (SSUs) were living quarters (LQs) within the EBs that had been randomly selected. A total of 10,428 LQs were selected from Malaysia’s EBs; 536 from urban and 333 from rural areas. Each EB randomly selects 12 LQs.

This study used a single proportion formula to calculate sample size (12). The sample size for the entire study, however, was based on the largest sample size required due to the survey consisting of several topics; with an error margin of 0.01 to 0.05; a 95% confidence interval; a design effect adjustment of 1.5 to 2.0 and a non-response rate of 35%. Based on the mentioned considerations, out of 10,428 LQs selected, 9,433 were eligible, but only 8,411 participants responded during the survey, with an 89.2% response rate. From the households, 29,606 were successfully interviewed (13) and 29,460 respondents were available for analysis. The sample size allocation to each state is proportionate to the population size in each state and federal territories in Malaysia (12, 13).

Geographically, NHMS 2015 targeted urban and rural Malaysians residing in non-institutionalised living quarters from all 13 states and 3 federal territories (13). The survey did not include populations living in institutions like hotels, hospitals and others (12). Furthermore, the Department of Statistics Malaysia conducted the selection of samples using the updated 2014 sampling frame (13). Interviews were carried out for respondents aged 13 years old and above. Meanwhile, for respondents below 13 years old, the parent or guardian responded to the interview on their behalf (by proxy). Structured questionnaires were used to collect data based on the scope of the survey. There were two questionnaires; face-to-face interviews and self-administered (12). The structured questionnaire was divided into 29 modules that covered various topics.

The data section used in this study mainly includes modules A, B, C, D and E, which contain information regarding household profiles, expenditure and selected NCDs. The module that requires clinical assessment was done by nurses (12). The complete survey quality control checks were done at various stages (12). At the planning stage, correct survey design, verified questionnaires and instruments, instructions, and standardised training to ensure quality, were adopted. The on-site quality check starts with verifying the identification (ID) of the selected LQs based on the Department of Statistics map. Field supervisors were responsible for supervising interviews, data collection and reviewing all completed questionnaires at the end of the survey to ensure data quality. The data processing staff checked all centrally submitted questionnaires. An eligibility check was integrated into the application, based on age or sex, to ensure the quality of data captured.

### Ethical Approval Evidence

A formal request was filed to the Director-General of the Ministry of Health to get the raw NHMS data for this investigation. Clearance was granted in March 2020. The IPH’s Biostatic Sector and Respiratory Data provides a collection of raw data in Statistical Package for the Social Sciences (SPSS) format, a data usage agreement and a letter from the Director-General. The research must also be registered with the National Medical Research Register (NMRR). This research registration is then reviewed by the National Clinical Research Centre (NCRC) and the Medical Research and Ethics Committee (MREC). The use of this data for this study has been approved by relevant governing bodies, including the MOH Malaysia’s Director-General and the Medical Research and Ethics Committee.

### Statistical Analysis

STATA/SE for Windows version 15.1 was used in a logistic regression analysis to investigate the influence of independent variables on household poverty. The independent variables in this study were age group, household size, strata, gender, educational level, occupation status, catastrophic payment and NCD. Forced entry (entre method) standard variable selection techniques were used to derive the logistic model. The forced entry method is more accurate than the stepwise method, which is less reliable (14). This method is used because it relies on good theoretical reasons for including the chosen predictors. Some academics claimed that forced entry is the only appropriate method for theory testing because stepwise procedures are impacted by random variation in the data (15). Thus, rarely give replicable results when the model is retested (15).

The forward, backward and stepwise approaches are called stepwise since they all rely on mathematical criteria for a computer selecting variables. Numerous scholars claimed stepwise removes many critical methodological decisions from the researcher’s hands. Furthermore, the danger of over-fitting is when excessive variables in the model contribute little to predicting the outcome and under-fitting (leaving out essential predictors) the model. For these reasons, stepwise methods should be avoided if possible (15); hence, the study used the enter method. Finally, this study used The Hosmer-Lemeshow test to assess the goodness of fit models. The Hosmer-Lemeshow values were determined to reflect the model’s fit: the higher the *P*-value indicates, the better the model’s fit. The model modified by Ismail and Sivadas (16) is a probability model with a binary dependent value that carries either 0 or 1; hence, logistic regression is selected.

In this research, NCDs refer to households with or without non-communicable illnesses. At the same time, the individual’s poverty status is assigned to 1 if they are below the poverty line and 0 if not. Malaysia’s poverty line is RM930 per month per individual (17). Given that the NHMS data is for 2015, the poverty line income (PLI) is calculated using the household income survey and basic amenities (HIS/BA) 2014 data to ensure consistency. ‘Catastrophic payment variable’ refers to when out-of-pocket health expenditure (OOPHE) reaches a certain percentage of household income or total spending (18). The catastrophic payment is calculated as a binary: 1 if the household suffers catastrophic payment, 0 otherwise. The thresholds chosen in this analysis are the two recommended by the United Nations Sustainable Development Goals (SDGs): 10% and 25% of total spending (19). This study employed the approach proposed by (18, 20).

## Results

### Descriptive Analysis

The sample is described in detail in Table 1. In 2015, 24.20 % of B40 households lived below the poverty level on average. Furthermore, 47.32 % of B40 household members had at least one NCD, with 1.13 % incurring catastrophic payments at the 15% threshold and 0.65 % at the 25% threshold. Most respondents (51.18 % and 52.73 %, respectively) came from rural areas and were female. On average, 34.96 % of B40 households were under 20 years old, while 29.56 % were between 40 years old and 64 years old. At the same time, 36.20 % and 18.59 % of respondents were from households with secondary education and worked as private workers. The results in Table 2 also indicate that B40 households with diabetes mellitus, hypertension and hypercholesterolemia yield a higher percentage than the M40 (middle-income earners) and T20 groups (highest-income earners).

## Logistic Regression Results

This section discusses the results of the logistic analysis. The poverty status as the dependent variable takes on the value of 1 if an individual is below Malaysia’s poverty line of RM930 (USD211) per month and 0 is non-poor. The essential variables in the model were NCDs, a binary variable with a value of 1 if a person came from a household with NCDs and 0 if otherwise. Catastrophic payment is if a person experiences catastrophic payment for a health issue. Other demographic and socio-economic factors are included in the models. Table 3 displays the logistic regression results, which included four models among the B40 households. Model 1 contains demographic and socio-economic factors and does not include health-related factors. Models 2 and 3 display NCD-related factors and catastrophic payments, respectively. Finally, Model 4 demonstrates a complete list of variables, including NCDs and catastrophic payments.

Table 3 indicates the odd ratios (OR), the standard error in parenthesis and the significant levels of 1%, 5% and 10%. An event is more likely to occur when its OR is greater than 1, while an OR less than 1 suggests that the event is less likely to occur as the predictor rises. The logistic regression shows that B40 households with at least one member reporting an NCD were significantly more likely to experience poverty than those B40 households without a chronic illness (OR = 1.07; 95% CI: 0.99, 1.15) (Table 3). Catastrophic health payments occurred for the B40s when their spending on health exceeded 25% of total expenditure, which dragged them to the poverty level than those with less spending (OR = 1.44; 95% CI: 0.96, 2.16). The elderly in B40 households were significantly more likely to experience poverty than a younger group (OR = 3.35; 95% CI: 2.95, 3.80).

The B40 households with no formal education were more likely to experience poverty than higher education families (OR = 1.59; 95% CI: 1.39, 1.82). Besides that, B40 households with unpaid work were more likely to experience poverty than B40 households with private employees or self-employed (OR = 10.20; 95% CI: 6.84, 15.20). Gender and catastrophic payments at a 10% threshold were statistically insignificantly associated with poverty (Table 3). At the same time, it is interesting to note that columns 2 and 4 in Table 4 show that B40 households with NCDs have a 1% rate of being poorer compared to B40 households without NCDs. The results stated that B40 households with a catastrophic payment threshold of 25% have a rate of 5.9% of becoming poor, compared to B40 households with a lower ratio of catastrophic payments.

The summary of the marginal effects (Table 4) shows that B40s aged 65 years old and over have a 20.7% of being poor compared to young people. Higher education results in a lower likelihood of poverty (by 2.7% for tertiary education). B40s with no formal education and primary level are 7.9% and 3.6% more likely to experience poverty than individuals with higher education. The occupation status coefficients convey that B40s with jobs are less prone to becoming poor than B40s who do not have stable jobs. The results also show that B40s with private workers and self-employed have 5.7% and 13.1% of becoming poor compared to unpaid workers and retirees, with 23.4% and 17.6% being poor, respectively. As mentioned earlier, health-related factors include B40 households that reported at least one NCD and catastrophic health payments at 10% and 25% thresholds.

### Robustness Check

A robustness check is a circumstance in which the researcher analyses how these regression coefficient estimates perform when the regression specification is often changed by eliminating or adding the independent variable (21). The fact that the coefficient does not change substantially proves they are robust (21). Table 5 displays the robustness check for logistic regression analysis findings for each independent variable. Some independent variables’ reference categories are adjusted, eliminated and added to test the model’s robustness, and investigate their influence. According to the findings, the direction and importance of the results are constant and do not alter significantly. The robustness of the model shows that the direction of the main variables (NCDs) was changed after adding the new variables.

## Discussion

B40 households with members diagnosed with at least one NCD face a greater poverty risk than those without NCDs. Alarmingly, NCDs in Malaysia are more prevalent among B40s aged 40 years old and above (6). Public hospitals offer minimal hospitalisation and medication fees, especially to the bottom population of the B40s. However, NCDs typically require expensive medical aid and equipment for the patient, burdening the B40 households. The results are consistent with past studies’ findings from Vietnam that found that households with at least one member reporting an NCD considerably face impoverishment than households without NCDs (22). A study in Mongolia also reported that households with a member affected by NCDs and multiple morbidities were more likely to experience medical impoverishment (23).

B40 households that incur catastrophic payments exceeding 25% of total income are likelier to experience poverty. For low-income B40 households lacking resources and insurance, making small payments for common illnesses can be financially devastating. Most of these B40 households pay for health needs with their income. They cannot afford a firm insurance policy. This puts them at risk of catastrophic payments, thus compounding their poverty. This finding is in line with a study from Iran, which indicated that a high incidence of catastrophic payments substantially increases the risk of household impoverishment. Most of these households belonged to the lower class of society (24). A study from Morocco reported that the poverty level enormously increases the likelihood of health expenditure becoming catastrophic (25).

Being elderly in a B40 household increases the probability of a household falling into poverty compared to younger groups. As age increases, the household becomes less able to work. The majority of older people are retired and have limited income. The opportunity to work is highly restricted, putting their finances at risk. Besides that, the older population also needs frequent access to healthcare for survival, as their age tends to be exposed to multiple health issues. This issue led to higher medical expenses for the elderly B40 group than for younger ones. The findings from previous studies showed older patients in China were more likely to experience post-treatment household impoverishment than their younger counterparts (26). A similar study in India mentioned that the larger the proportion of the elderly in a household, the more likely they are to descend into poverty (27).

The current analysis results indicate that the lower the education level, the higher the odds of B40 households experiencing poverty. B40 households without formal education are more likely to be in poverty than households with formal education. B40 households with low educational levels engaged in low-skill and less productive sectors. Households with a low level of education tend to have limited access to employment opportunities, social facilities and services. This situation will lead them to have low earnings and experience poverty. The results support previous research conducted in South Africa, which found that there is a probability of being poor with the household head’s education level (28). Previous research in Malaysia found that education levels were statistically significant in determining poverty among the elderly (29).

The B40 households without a stable income, such as unpaid workers and retirees, are at a higher risk of poverty; compared to the B40 households that work as private employees or are self-employed. A working B40 is less likely to be poor than a B40 who does not have a stable job. Private workers or self-employed individuals have a fixed income to support their cost of living. They are also covered by effective health protection schemes, such as employer-provided and personal insurance, to protect them from unavoidable expenses. Unpaid workers and retirees with minimal income are more prone to become poor quickly. The current finding is supported by similar studies in South Africa, where the type of employment status of the household head has the probability of being poor (28). Furthermore, study results from Indonesia stated that the increase in unemployment in Jambi Province would likely affect poverty in Jambi Province (30).

Examining the impoverishing effects of NCDs and catastrophic spending will reflect how the healthcare system responds to NCDs, particularly regarding household financial risk protection and the adequacy of healthcare service provision. It also provides the opportunity to reveal possible gaps in the system, paving the way for future research to explore new strategies and solutions for effective NCDs management. This paper is a fresh approach to relating health factors with poverty; through incidences of catastrophic payments caused by NCDs for low-income households. While several studies estimate the prevalence of catastrophic payments, they do not objectively relate to poverty (24, 31). These contributions should positively impact homes, society and the country. It will give information that may help improve policy development and support targeted policy approaches in public health, household welfare and healthcare access.

This survey uses a large nationally representative sample, which provides reliable and valid data for examining the impact of NCDs among low-income adults in Malaysia. The limitation of this study is the cross-sectional nature of the survey, which prevents the ID of causal relationships between identified factors and poverty. There is also a possibility of reverse causality. The study could only observe the associations between NCDs and poverty, and no causal relationships could be determined. Thus, we need data over several years to understand the causal effect of NCDs on various outcomes. Despite the limitations, this cross-sectional method can estimate and analyse the prevalence of outcomes because the sample is taken from the whole population. Moreover, this data could also capture many outcomes, factors and associated characteristics that can be assessed.

## Conclusion

Given the scarcity of research on chronic NCDs in Malaysia, this study seeks to examine the impacts of NCDs on household poverty among the B40 group in Malaysia. The dependent variables were binary and the final objective of the study used a logistic regression model. The study classified the causes of poverty in each group of variables as demographic, socio-economic and health characteristics. The sample size of the B40 income group was 18,616 respondents. About 24.20% of B40 households who participated in the NHMS 2015 survey (4,506 individuals) lived in poverty in 2015. According to the study, Malaysia has a high rate of diagnosed NCDs, particularly among the B40s, with 47.32% of B40 households (8,810 individuals) diagnosed with at least one NCD. Most respondents burdened by catastrophic payments account for 2% of the sample size.

This research suggests that households with NCDs, having to spend catastrophic payments at 25% of household resources, the elderly, those with little formal education and unpaid workers are more likely to be poor. These characteristics were significant causes of poverty, implying that strategies to alleviate poverty should not disregard these health variables. In this thesis, a new study was conducted relating poverty to catastrophic payments and NCDs. This study is a radical shift from the traditional approach to modelling poverty. The research has made an essential contribution to the literature by conducting an in-depth investigation and analysis of the indicator. Hopefully, authorities will use this study’s conclusions as a guideline to identify possible strategies, to reduce the prevalence of NCDs and catastrophic payments, through appropriate preventative action and plans.

## Figures and Tables

**Table 1:** Descriptive analysis (*N* **=** 18,616)

	*n*	%
Age (years old)		
0–19	6,509	35.0
20–39	4,629	24.9
40–64	5,502	29.6
65 above	1,976	10.6
Gender		
Female	9,816	52.7
Male	8,800	47.3
Strata		
Rural	9,527	51.2
Urban	9,089	48.8
Log household size (1–14)		
Education		
No formal education	1,555	8.4
Primary	6,170	33.1
Secondary	6,739	36.2
Tertiary	1,576	8.5
Unclassified	2,576	13.8
Occupation		
Government servant	726	3.9
Private employee	3,461	18.6
Self employed	2,527	13.6
Unpaid worker	2,592	13.9
Retiree	544	2.9
Other	8,766	47.1
Catastrophic payment		
CAT10	304	1.6
CAT25	121	0.65
Poor	4,506	24.2
NCD	8,810	47.3

Notes: SD = standard deviation; CAT10 = household incur catastrophic payments more than 10% of households’ resources; CAT25 = household incur catastrophic payments more than 25% of households’ resources

**Table 2:** Diabetes, hypertension, and hypercholesterolaemia prevalence among income groups

	B40 (%)	M40 (%)	T20 (%)
Diabetes	2,917 (9.9)	1,000 (3.4)	312 (1.1)
Hypertension	5,043 (17.1)	1,615 (5.5)	567 (1.9)
Hypercholesterolaemia	6,742 (22.9)	2,809 (9.5)	963 (3.3)

Notes: B40 = bottom income earners; M40 = middle income earners; T20 = highest income earners

**Table 3:** Logistic regression result

Models	1			2			3			4		
Factors	*b*	OR (95% CI)	*P*-value	*b*	OR (95% CI)	*P*-value	*b*	OR (95% CI)	*P*-value	*b*	OR (95% CI)	*P*-value
Age group (years old) (0–19)^a^												
20–39	0.52	1.68 (1.45, 1.93)	< 0.001	0.52	1.69 (1.46, 1.95)	< 0.001	0.51	1.67 (1.44, 1.93)	< 0.001	0.52	1.68 (1.45, 1.94)	< 0.001
40–64	0.78	2.18 (1.92, 2.48)	< 0.001	0.78	2.19 (1.93, 2.48)	< 0.001	0.78	2.18 (1.92, 2.47)	< 0.001	0.78	2.18 (1.92, 2.48)	< 0.001
65 above	1.21	3.35 (2.95, 3.80)	< 0.001	1.20	3.33 (2.93, 3.78)	< 0.001	1.20	3.33 (2.93, 3.78)	< 0.001	1.20	3.31 (2.92, 3.76)	< 0.001
Gender (Female)	0.03	1.03 (0.95, 1.12)	0.438	0.03	1.03 (0.95, 1.12)	0.463	0.03	1.03 (0.95, 1.12)	0.430	0.03	1.03 (0.95, 1.12)	0.454
Strata (Urban)	−0.15	0.86 (0.80, 0.93)	< 0.001	−0.15	0.86 (0.80, 0.93)	< 0.001	−0.15	0.86 (0.80, 0.93)	< 0.001	−0.15	0.86 (0.80, 0.93)	< 0.001
Household size	−0.86	0.42 (0.38, 0.47)	< 0.001	−0.85	0.43 (0.39, 0.48)	< 0.001	−0.86	0.42 (0.38, 0.47)	< 0.001	−0.84	0.43 (0.39, 0.48)	< 0.001
Education (Secondary)^a^												
No formal education	0.46	1.59 (1.39, 1.82)	< 0.001	0.46	1.59 (1.39, 1.82)	< 0.001	0.46	1.59 (1.39, 1.82)	< 0.001	0.46	1.59 (1.38, 1.82)	< 0.001
Primary	0.22	1.25 (1.14, 1.37)	< 0.001	0.22	1.25 (1.14, 1.37)	< 0.001	0.22	1.25 (1.14, 1.37)	< 0.001	0.22	1.25 (1.14, 1.37)	< 0.001
Tertiary	−0.18	0.84 (0.71, 0.99)	0.034	−0.18	0.83 (0.71, 0.99)	0.033	−0.18	0.84 (0.71, 0.99)	0.34	−0.18	0.83 (0.71, 0.98)	0.032
Unclassified	0.11	1.12 (0.98, 1.28)	0.103	0.11	1.11 (0.97, 1.27)	0.118	0.11	1.12 (0.99, 1.28)	0.111	0.10	1.11 (0.97, 1.27)	0.127
Occupation (Government servant)^a^												
Private employee	0.99	2.69 (1.80, 4.02)	< 0.001	0.99	2.68 (1.79, 4.01)	< 0.001	0.99	2.69 (1.80, 4.03)	< 0.001	0.99	2.68 (1.79, 4.01)	< 0.001
Self employed	1.67	5.32 (3.57, 7.94)	< 0.001	1.67	5.30 (3.55, 7.90)	< 0.001	1.67	5.33 (3.57, 7.95)	< 0.001	1.67	2.68 (3.56, 7.91)	< 0.001
Unpaid worker	2.32	10.20 (6.84,15.20)	< 0.001	2.32	10.18 (6.83, 15.18)	< 0.001	2.32	10.17 (6.82, 15.16)	< 0.001	2.32	10.16 (6.82, 15.14)	< 0.001
Retiree	1.99	7.30 (4.73, 11.25)	< 0.001	1.98	7.22 (4.68, 11.13)	< 0.001	1.99	7.30 (4.73, 11.25)	< 0.001	1.98	7.22 (4.68, 11.13)	< 0.001
Others	2.69	14.75 (9.91, 21.97)	< 0.001	2.69	14.63 (9.82, 21.80)	< 0.001	2.69	14.74 (9.90, 21.95)	< 0.001	2.68	14.62 (9.82, 21.77)	< 0.001
NCD				0.07	1.07 (0.99, 1.15)	0.081				0.06	1.07 (0.99, 1.15)	0.087
Catastrophic payment												
CAT10							0.14	1.15 (0.88, 1.51)	0.318	0.13	1.14 (0.87, 1.50)	0.334
CAT25							0.37	1.44 (0.96, 2.16)	0.076	0.36	1.44 (0.96, 2.15)	0.078
*R* ^2^	0.10			0.10			0.10			0.10		
Hosmer-Lemeshow test	*χ^2^* (8) = 12.70, *P* = 0.123			*χ^2^* (8) = 12.73, *P* = 0.122			*χ^2^* (8) = 13.68, *P* = 0.090			*χ^2^* (8) = 13.09, *P* = 0.109		
Observations	18,616			18,616			18,616			18,616		

Notes: *b* = coefficient; OR = odd ratios; CI = confidence interval; NCD = non-communicable diseases; CAT10 = household incur catastrophic payments more than 10% of households’ resources; CAT25 = household incur catastrophic payments more than 25% of households’ resources; 1 = first model; 2 = second model; 3 = third model; 4 = fourth model;

a Reference

**Table 4:** Impact of demographic, socioeconomic, NCD and catastrophic payment factors on poverty-marginal effects

Models	1		2		3		4	
				
Factors	Dydx (95% CI)	*P*-value	Dydx (95% CI)	*P*-value	Dydx (95% CI)	*P*-value	Dydx (95% CI)	*P*-value
Age group (years old) (0–19)^a^								
20–39	0.08 (0.06, 0.10)	< 0.001	0.09 (0.06, 0.10)	< 0.001	0.09 (0.06, 0.10)	< 0.001	0.08 (0.06, 0.10)	< 0.001
40–64	0.13 (0.10, 0.14)	< 0.001	0.13 (0.10, 0.14)	< 0.001	0.13 (0.10, 0.14)	< 0.001	0.13 (0.10, 0.14)	< 0.001
65 above	0.21 (0.18, 0.23)	< 0.001	0.21 (0.18, 0.23)	< 0.001	0.21 (0.18, 0.23)	< 0.001	0.21 (0.18, 0.23)	< 0.001
Gender (Female)	0.01 (−0.01, 0.02)	0.454	0.01 (−0.01, 0.02)	0.454	0.01 (−0.01, 0.02)	0.454	0.01 (−0.01,0.02)	0.454
Strata (Urban)	−0.02 (−0.04, −0.01)	< 0.001	−0.02 (−0.04, −0.01)	< 0.001	−0.02 (−0.04, −0.01)	< 0.001	−0.02 (−0.04, −0.01)	< 0.001
Household Size	−0.14 (−0.15, −0.12)	< 0.001	−0.14 (−0.15, −0.12)	< 0.001	−0.14 (−0.15, −0.12)	< 0.001	−0.14 (−0.15, −0.12)	< 0.001
Education (Secondary)^a^								
No formal education	0.08 (0.05, 0.10)	< 0.001	0.08 (0.05, 0.10)	< 0.001	0.08 (0.05, 0.10)	< 0.001	0.08 (0.05, 0.10)	< 0.001
Primary	0.04 (0.02, 0.05)	< 0.001	0.04 (0.02, 0.05)	< 0.001	0.04 (0.02, 0.05)	< 0.001	0.04 (0.02, 0.05)	< 0.001
Tertiary	−0.03 (−0.05, −0.003)	0.027	−0.03 (−0.05, −0.003)	0.027	−0.03 (−0.05, −0.003)	0.027	−0.03 (−0.05, −0.003)	0.027
Unclassified	0.02 (−0.005, −0.04)	0.130	0.02 (−0.005, 0.04)	0.130	0.02 (−0.005, 0.04)	0.130	0.02 (−0.005, 0.04)	0.130
Occupation (Government servant)^a^								
Private employee	0.06 (0.04, 0.07)	< 0.001	0.06 (0.04, 0.07)	< 0.001	0.06 (0.04, 0.07)	< 0.001	0.06 (0.04, 0.07)	< 0.001
Self employed	0.13 (0.11, 0.15)	< 0.001	0.13 (0.11, 0.15)	< 0.001	0.13 (0.11, 0.15)	< 0.001	0.13 (0.11, 0.15)	< 0.001
Unpaid worker	0.23 (0.21, 0.26)	< 0.001	0.23 (0.21, 0.26)	< 0.001	0.23 (0.21, 0.26)	< 0.001	0.23 (0.21, 0.26)	< 0.001
Retiree	0.18 (0.14, 0.21)	< 0.001	0.18 (0.14, 0.21)	< 0.001	0.18 (0.14, 0.21)	< 0.001	0.18 (0.14, 0.21)	< 0.001
Others	0.31 (0.29, 0.33)	< 0.001	0.31 (0.29, 0.33)	< 0.001	0.31 (0.29, 0.33)	< 0.001	0.31 (0.29, 0.33)	< 0.001
NCD			0.01 (−0.002, 0.02)	0.087			0.01 (−0.002, 0.02)	0.087
Catastrophic payment								
CAT10					0.02 (−0.02, 0.07)	0.334	0.02 (−0.02, 0.07)	0.334
CAT25					0.06 (−0.01, 0.12)	0.078	0.06 (−0.01, 0.12)	0.078

Notes: CI = confidence interval; NCD = non-communicable diseases; CAT10 = household incur catastrophic payments more than 10% of households’ resources; CAT25 = household incur catastrophic payments more than 25% of households’ resources; 1 = first model; 2 = second model; 3 = third model; 4 = fourth model;

a Reference

**Table 5:** Robustness check

Models	1			2			3			4		
				
Factors	*b*	OR (95% CI)	*P*-value	*b*	OR (95% CI)	*P*-value	*b*	OR (95% CI)	*P*-value	*b*	OR (95% CI)	*P*-value
Age group (years old) (40–64)^a^												
0–19	−0.77	0.46 (0.41, 0.53)	< 0.001	−1.14	0.32 (0.27, 0.37)	< 0.001	−0.77	0.46 (0.41, 0.53)	< 0.001	−1.14	0.32 (0.27, 0.37)	< 0.001
20–39	−0.30	0.74 (0.66, 0.83)	< 0.001	−0.46	0.63 (0.54, 0.74)	< 0.001	−0.30	0.74 (0.66, 0.83)	< 0.001	−0.46	0.63 (0.54, 0.73)	< 0.001
65 above	0.48	1.62 (1.43, 1.83)	< 0.001	0.56	1.74 (1.51, 2.02)	< 0.001	0.48	1.62 (1.43, 1.84)	< 0.001	0.56	1.74 (1.50, 2.02)	< 0.001
Gender (Male)	−0.05	0.95 (0.88, 1.03)	0.207	−0.03	0.97 (0.88, 1.07)	0.511	−0.06	0.94 (0.87, 1.02)	0.160	−0.04	0.96 (0.88, 1.06)	0.442
Strata (Rural)	0.18	1.20 (1.11, 1.30)	< 0.001	0.15	1.16 (1.05, 1.27)	0.002	0.18	1.20 (1.11, 1.29)	< 0.001	0.14	1.15 (1.05, 1.27)	0.003
Household size	−0.86	0.42 (0.38, 0.46)	< 0.001	−0.84	0.43 (0.37, 0.50)	< 0.001	−0.86	0.42 (0.38, 0.46)	< 0.001	−0.84	0.43 (0.37, 0.50)	< 0.001
Education (Secondary)^a^												
No formal education	0.33	1.39 (1.21, 1.60)	< 0.001	0.28	1.32 (1.11, 1.56)	0.001	0.33	1.39 (1.20, 1.59)	< 0.001	0.27	1.31 (1.10, 1.56)	0.002
Primary	0.17	1.19 (1.08, 1.31)	< 0.001	0.13	1.13 (1.01, 1.27)	0.035	0.17	1.19 (1.08, 1.31)	< 0.001	0.13	1.14 (1.01, 1.28)	0.034
Tertiary	−0.16	0.86 (0.72, 1.01)	0.068	−0.21	0.81 (0.66, 0.99)	0.045	−0.14	0.87 (0.74, 1.03)	0.114	−0.19	0.83 (0.67, 1.02)	0.070
Unclassified	0.07	1.08 (0.94, 1.23)	0.296	0.17	1.18 (1.01, 1.39)	0.042	0.07	1.07 (0.94, 1.23)	0.299	0.16	1.18 (1.00, 1.38)	0.044
Occupation (Government servant)^a^												
Private employee	0.92	2.52 (1.68, 3.77)	< 0.001	0.58	1.80 (1.13, 2.84)	0.013	0.91	2.49 (1.66, 3.73)	< 0.001	0.58	1.79 (1.13, 2.83)	0.013
Self employed	1.63	5.10 (3.42, 7.62)	< 0.001	1.15	3.17 (2.01, 5.00)	< 0.001	1.62	5.07 (3.40, 7.58)	< 0.001	1.16	3.18 (2.01, 5.01)	< 0.001
Unpaid worker	2.27	9.72 (6.52,14.50)	< 0.001	1.76	5. 83 (3.70, 9.20)	< 0.001	2.28	9.79 (6.56, 14.61)	< 0.001	1.78	5.912 (3.75, 9.33)	< 0.001
Retiree	1.96	7.07 (4.58, 10.92)	< 0.001	1.52	4.56 (2.80, 7.42)	< 0.001	1.97	7.19 (4.66, 11.10)	< 0.001	1.54	4.65 (2.86, 7.58)	< 0.001
Other	2.66	14.29 (9.59, 21.31)	< 0.001	2.32	10.13 (6.46, 15.88)	< 0.001	2.67	14.32 (9.60, 21.35)	< 0.001	2.32	10.23 (6.52, 16.04)	< 0.001
Ethnic												
Malay	−0.66	0.52 (0.46, 0.58)	< 0.001	−0.62	0.54 (0.47, 0.62)	< 0.001	−0.67	0.51 (0.46, 0.57)	< 0.001	−0.63	0.53 (0.46, 0.61)	< 0.001
Chinese	−0.26	0.78 (0.67, 0.90)	< 0.001	−0.28	0.76 (0.63, 0.90)	0.002	−0.27	0.77 (0.66, 0.89)	< 0.001	−0.29	0.75 (0.63, 0.89)	0.001
Indian	−0.48	0.62 (0.52, 0.74)	< 0.001	−0.42	0.66 (0.53, 0.81)	< 0.001	−0.48	0.62 (0.52, 0.74)	< 0.001	−0.42	0.66 (0.53, 0.82)	< 0.001
Other	−0.31	0.73 (0.61, 0.87)	< 0.001	−0.34	0.71 (0.57, 0.89)	< 0.001	−0.32	0.72 (0.61, 0.86)	< 0.001	−0.36	0.70 (0.56, 0.88)	0.002
NCD				0.10	1.10 (0.94, 1.30)	0.242				0.10	1.10 (0.93, 1.30)	0.256
Diabetes				0.01	1.01 (0.91, 1.13)	0.801				0.02	1.02 (0.91, 1.14)	0.734
Hypertension				0.24	1.28 (1.14, 1.42)	< 0.001				0.24	1.27 (1.14, 1.42)	< 0.001
Hyper cholesterol				0.01	1.01 (0.90, 1.15)	0.823				0.01	1.01 (0.90, 1.15)	0.824
LogOOP							−0.07	0.94 (0.91, 0.96)	< 0.001	−0.07	0.94 (0.90, 0.96)	< 0.001
Catastrophic payment												
CAT10							0.37	1.44 (1.08, 1.92)	0.013	0.47	1.60 (1.13, 2.27)	0.008
CAT25							0.57	1.78 (1.17, 2.72)	0.008	0.46	1.59 (0.94, 2.69)	0.082
*R* ^2^	0.11			0.13			0.11			0.13		

Notes: *b* = coefficient; OR = odd ratios; CI = confidence interval; NCD = non-communicable diseases; CAT10 = household incur catastrophic payments more than 10% of households’ resources; CAT25 = household incur catastrophic payments more than 25% of households’ resources; 1 = first model; 2 = second model; 3 = third model; 4 = fourth model;

a Reference
